# Supplementary material for: Black carbon footprint of human presence in Antarctica
Source: Nat Commun. 2022 Feb 22;13:984. doi: 10.1038/s41467-022-28560-w (PMC8863810; doi:10.1038/s41467-022-28560-w)
Supplement: Supplementary file 1 — Supplementary Information [file 41467_2022_28560_MOESM1_ESM.pdf]

# Supplementary Information

## Black Carbon Footprint of Human Presence in Antarctica


Raúl R. Cordero[1], Edgardo Sepúlveda[1], Sarah Feron[1,2,*], Alessandro Damiani[3,*], Francisco Fernandoy[4], Steven Neshyba[5], Penny M. Rowe[6], Valentina Asencio[7], Jorge Carrasco[8], Juan A. Alfonso[9], Pedro Llanillo[10], Paul Wachter[11], Gunther Seckmeyer[12], Marina Stepanova[1,*], Juan M. Carrera[1], Jose Jorquera[1], Chenghao Wang[13], Avni Malhotra[14], Jacob Dana[15], Alia L. Khan[15,16], Gino Casassa[8]

1 Universidad de Santiago de Chile. Av. Bernardo O'Higgins 3363, Santiago, Chile.
2 University of Groningen, 8911 CE, Leeuwarden, The Netherlands
3 Center for Environmental Remote Sensing, Chiba University, 1-33 Yayoicho, Inage Ward, Chiba, 263-8522, Japan
4 Universidad Andrés Bello, Quillota 980, Viña del Mar, Chile.
5 University of Puget Sound, Department of Chemistry, Tacoma, WA, USA
6 NorthWest Research Associates, Redmond, WA, USA
7 Select Carbon Pty Ltd, 562 Wellington Street, Perth WA 6000, Australia.
8 University of Magallanes, Av. Manuel Bulnes 1855, Punta Arenas, Chile.
9 Instituto Venezolano de Investigaciones Cientificas (IVIC), Apartado 20632, Caracas, Venezuela.
10 Alfred Wegener Institute (AWI), Am Handelshafen 12, 27570 Bremerhaven, Germany
11 German Aerospace Center (DLR), German Remote Sensing Data Center (DFD), Wessling, Germany
12 Leibniz Universität Hannover, Herrenhauser Strasse 2, Hannover, Germany.
13 Department of Earth System Science, Stanford University, Stanford, CA, 94305, USA.
14 University of Zurich, Winterthurerstrasse 190, 8057 Zürich, Switzerland
15 Western Washington University, 516 High St, Bellingham, WA 98225, USA
16 National Snow and Ice Data Center, Cooperative Institute for Research in Environmental Sciences, University of Colorado – Boulder, Boulder, CO, USA

* Corresponding Authors

Sarah Feron
s.c.feron@rug.nl
Alessandro Damiani
damiani@chiba-u.jp
Marina Stepanova
marina.stepanova@usach.cl

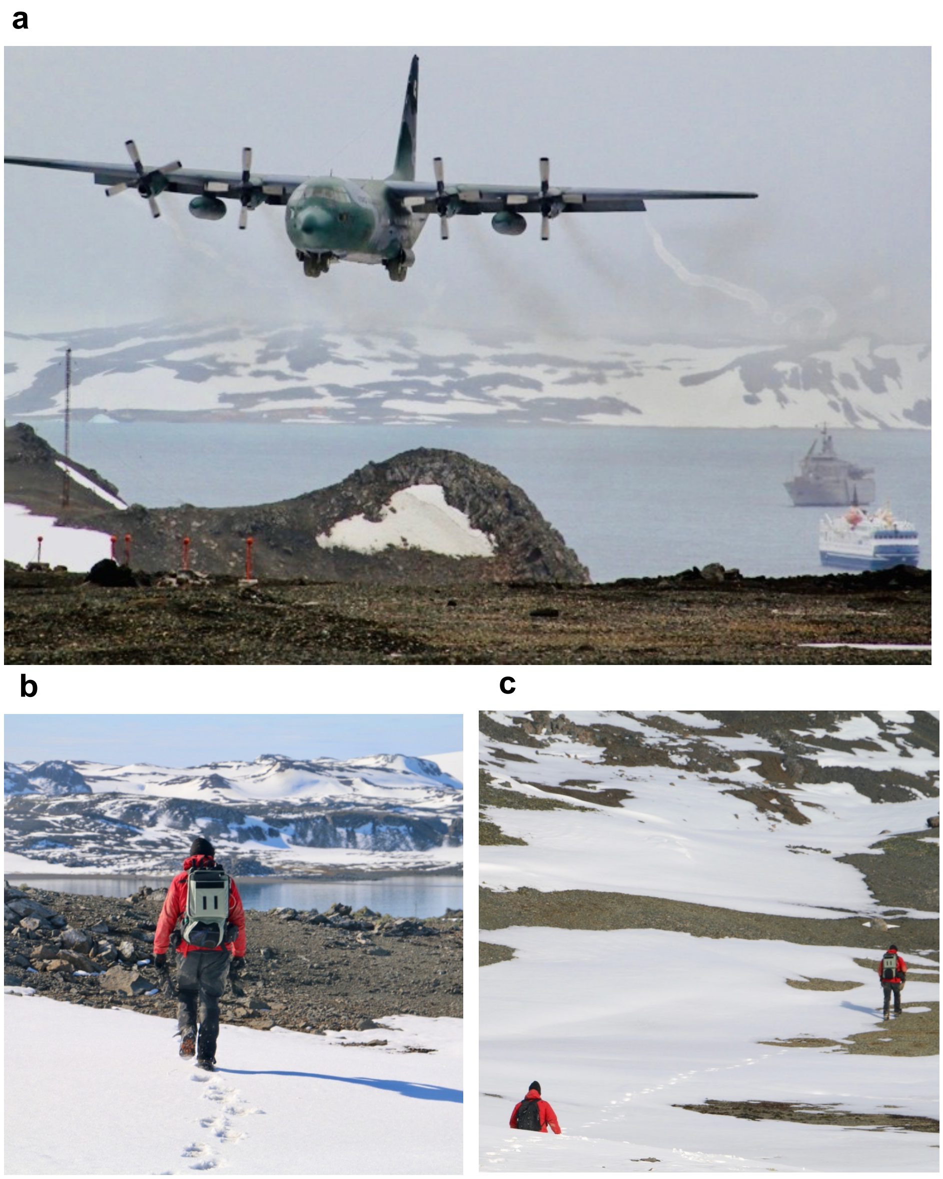


**Supplementary Figure 1.**

a) Four-engine aircraft about to land on King George Island (62°S, South Shetland Islands). Research stations operated by Argentina, Brazil, Chile, China, South Korea, Peru, Poland, Russia, and Uruguay are also located on the island[33], where Chile operates one of the busiest Antarctic airfields. Although we did sample on King George Island, we avoided sampling at the airfield because we aimed to assess the influence of light-absorbing impurities on the albedo of more representative areas.

b-c) We took snow samples about 3-km South of the airfield on King George Island that are likely more representative on a regional scale. Samples were taken at 8 sites on King George Island (Table 1), always several kilometers away from the airfield.

Photographs taken by the authors.

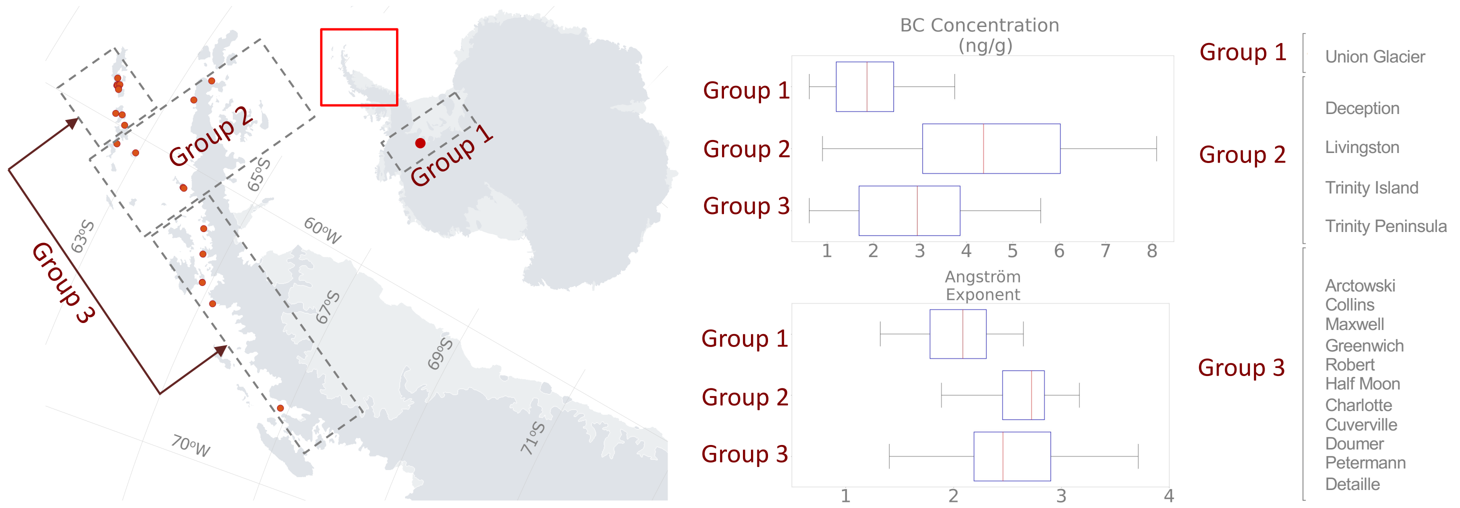


**Supplementary Figure 2**

Site groupings. In each box, the central mark (red stripe) indicates the median, and the edges indicate the 25th and 75th percentiles. The whiskers extend to the maximum and minimum data excluding outliers. Measurements of the Black Carbon (BC) concentration and the Ångström exponent at these sites were subjected to an Analysis of Variance (ANOVA) (Table S1) and a Tukey's Honestly Significant Difference Test (Table S2).

Plots were generated by using Python's Matplotlib Library[58].

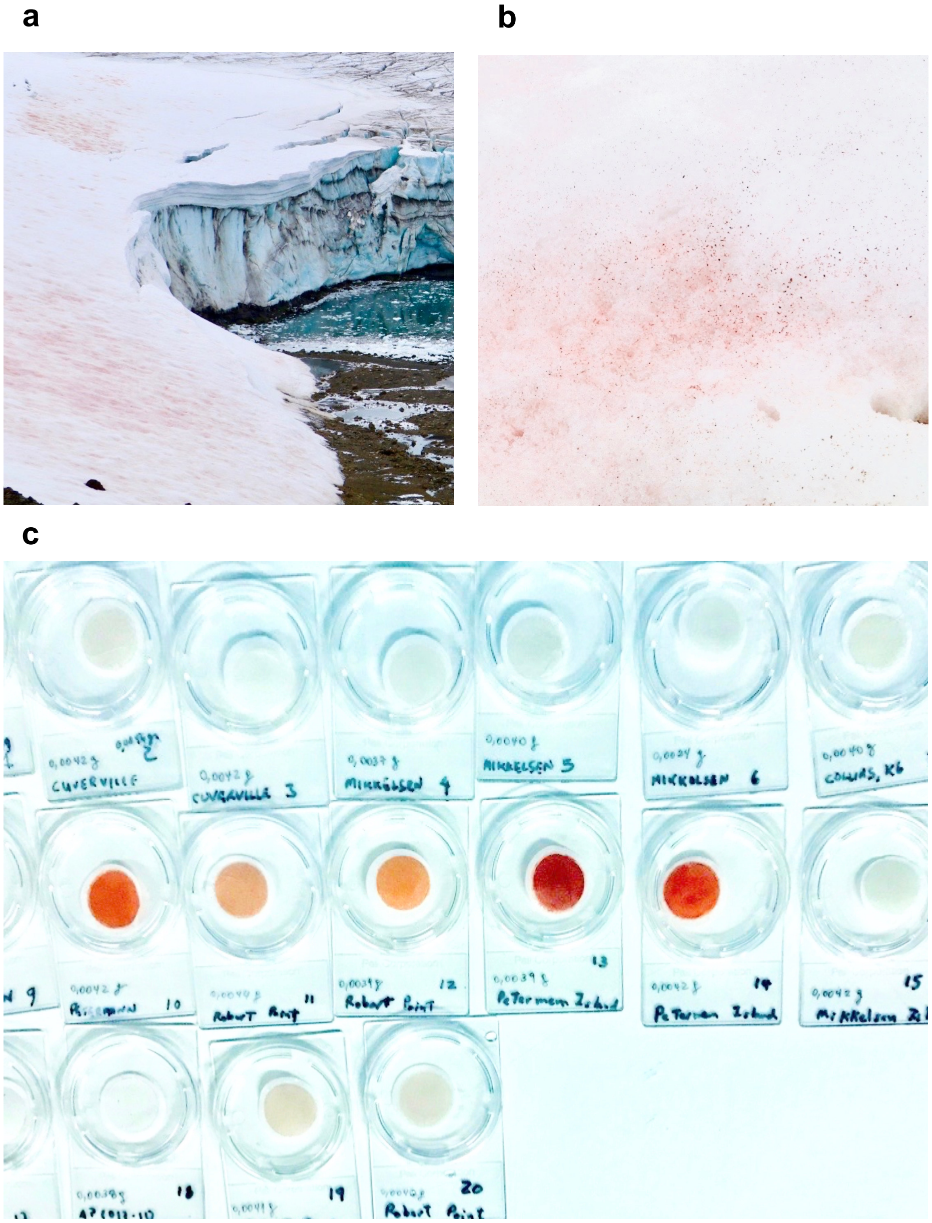


**Supplementary Figure 3**

a) Snow algal field on Collins Glacier (King George Island);

b) Closer look at the snow surface shown in a);

c) Although we avoided sampling on algal fields, the footprint of the algal pigment appeared on some filters corresponding to samples from Petermann Island, Robert Island, King George Island, and Doumer Island. We measured the absorption Ångström exponent ($a$) corresponding to these samples (Fig. S2). However, due to uncertainties regarding the apportioning of light absorption to Black Carbon (BC) and algae contributions, the BC concentration determined from these samples was not included in Figs. 2-3.

Photographs taken by the authors.

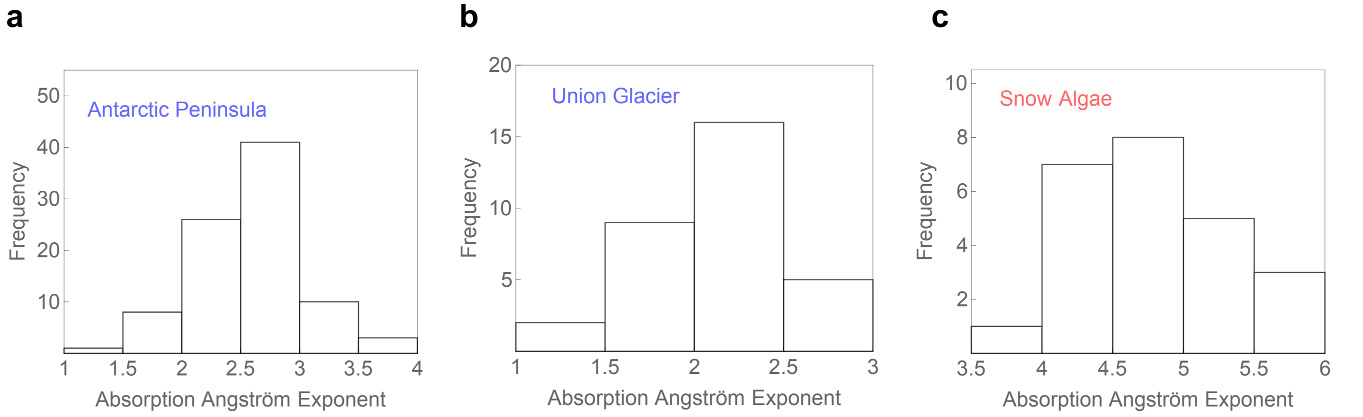


**Supplementary Figure 4**

Frequency distributions of the measurements of absorption Ångström exponent ($\alpha$) in snow samples. The (mean ± standard deviations) are indicated below.

a) Antarctic Peninsula and associated archipelagos ($\alpha$=2.5±0.5) (excepting samples where algae presence was apparent);
b) Union Glacier ($\alpha$=2.0±0.3);
c) Samples with snow algae ($\alpha$=4.9±0.6).

Plots were generated by using Python's Matplotlib Library[58].

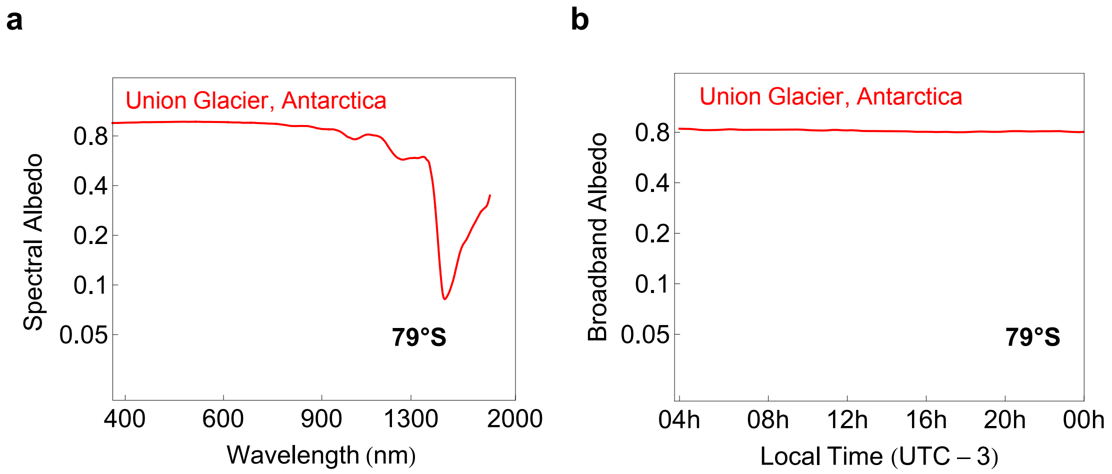


**Supplementary Figure 5**

Ground-based measurements of the albedo carried out at Union Glacier Camp (see Table 1 for details).

a) Spectral albedo measured in December 2015,
b) Broadband (SW) albedo measured in December 2017.

Plots were generated by using Python's Matplotlib Library[58].

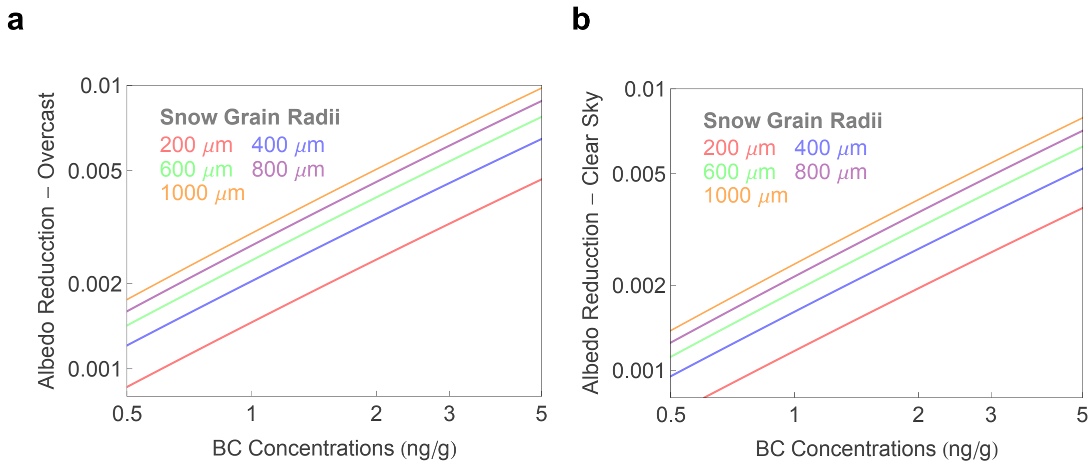


**Supplementary Figure 6**

Broadband albedo reduction due to given Black Carbon (BC) concentrations, according to the parameterization by Dang et al.[41].

a) Albedo reductions under overcast conditions ($\Delta A_{cloudy}$);
b) Albedo reductions under cloudless conditions ($\Delta A_{clear}$).

Plots were generated by using Python's Matplotlib Library[58].

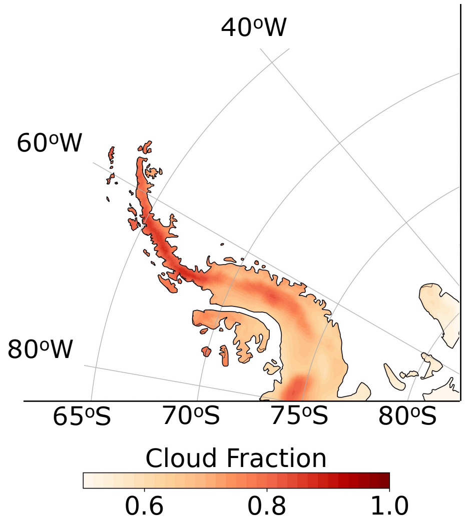


**Supplementary Figure 7**

Cloud fraction (*CF*) averaged for December, January and February (DJF) over the period 1981-2019. Data from ERA5 reanalysis[46] were used.

Plots were generated by using Python's Matplotlib Library[58].

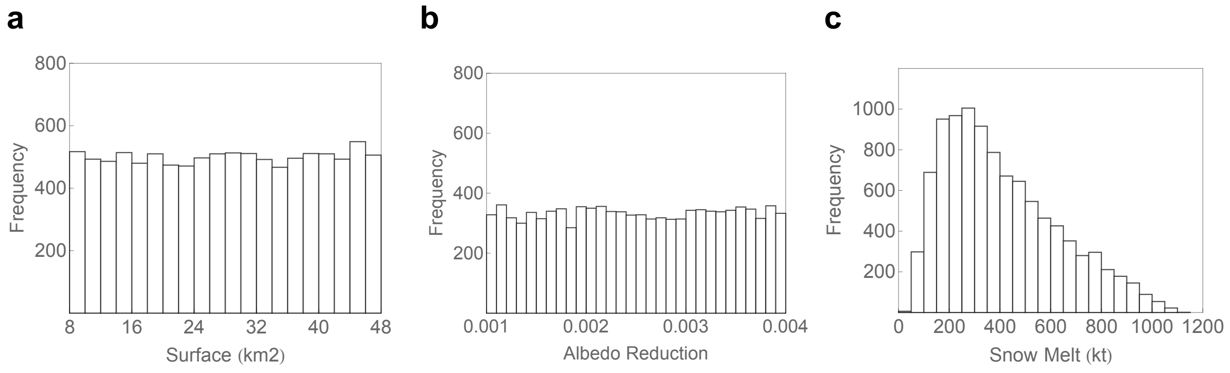


**Supplementary Figure 8**

Monte Carlo simulations for estimating the snow that melts sooner due to Black Carbon (BC) deposition from research stations on King George Island.

a) BC-impacted area. The histogram was built up by randomly generating surface values over the range 8-48 km2.

b) Albedo reductions ($\Delta A$). The histogram was built up by randomly generating $\Delta A$ values over the range 0.001-0.004.

c) Snow that melts sooner. Each value in the histogram was rendered by Eq. (13), using as inputs the values in a) and b). The mean and the standard deviations of these simulations were used for estimating the snow that melts sooner every summer due to BC deposition from local sources on King George Island: 0.4±0.2 Mt, which translates to about 0.6±0.3 kt of snow per bed; there are 11 research stations with a total of 700 beds on King George Island[33].

Plots were generated by using Python's Matplotlib Library[58].

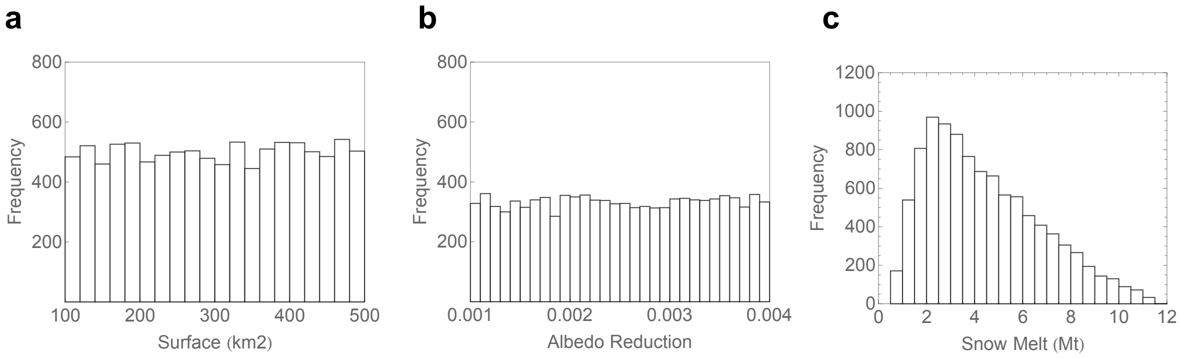


**Supplementary Figure 9**

Monte Carlo simulations for estimating the snow that melts sooner due to Black Carbon (BC) emissions associated with tourism in the Antarctic Peninsula and associated archipelagos.

a) BC-impacted area. The histogram was built up by randomly generating surface values over the range 100-500 km2.

b) Plausible albedo reductions ($\Delta A$). The histogram was built up by randomly generating $\Delta A$ values over the range 0.001-0.004.

c) Snow that melts sooner due to BC deposition associated with tourism in the Antarctic Peninsula and associated archipelagos. Each value in the histogram was rendered by Eq. (13), using as inputs the values in a) and b). The mean and the standard deviations of these simulations were used for estimating the snow that melts sooner every summer due to BC emissions associated with tourism in the Antarctic Peninsula and associated archipelagos: $4.4\pm2.3$ Mt, which translates to about $83\pm43$ tons per visitor; an average of 53,000 tourists visited the region from the season 2016-17 to the season 2019-2020[36].

Plots were generated by using Python’s Matplotlib Library[58].

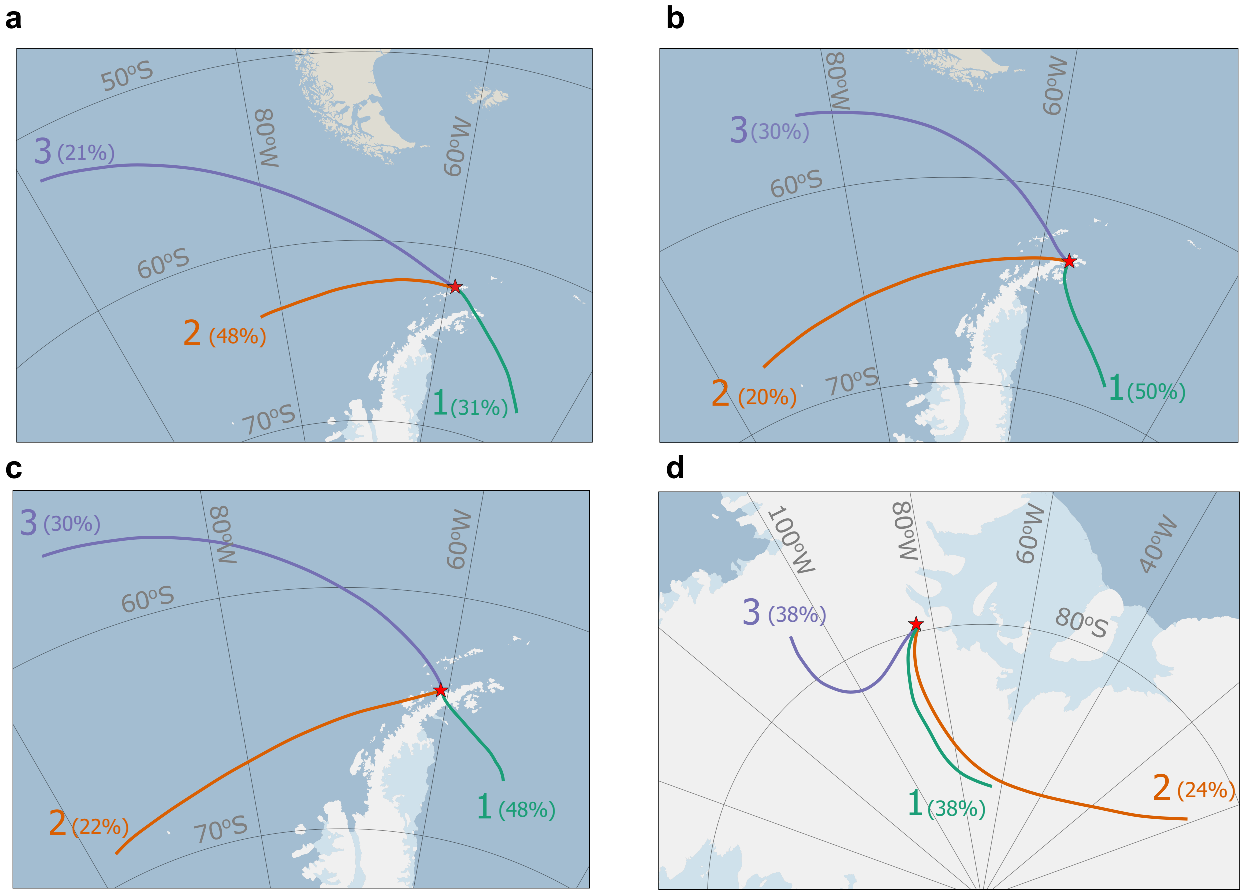


**Supplementary Figure 10**

Clusters of 72-h backward trajectories for December, January and February (DJF) days over the period 2010-2020.

a) Collins Glacier (Dome), King George Island;
b) Hope Bay, Trinity Peninsula;
c) Mikkelsen Harbor, Trinity Island;
d) Union Glacier, Ellsworth Mountains.

Back-trajectory analysis suggests that long-range transport (from Patagonia, for example) has only a minor role as a source of light-absorbing aerosols in Antarctica.

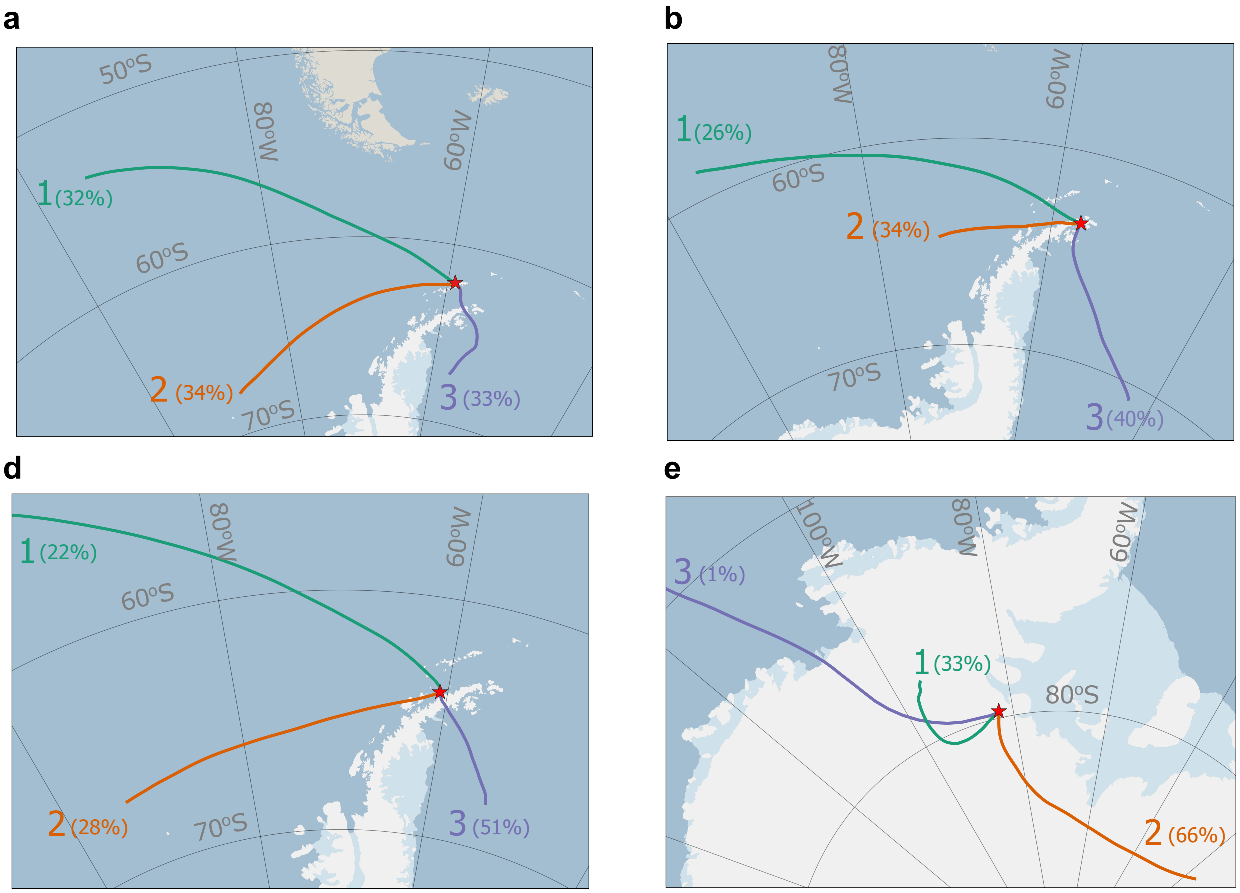


**Supplementary Figure 11**

Clusters of 72-h backward trajectories for December, January and February (DJF) days over the 3-month period before sampling.

a) Collins Glacier (Dome), King George Island;
b) Hope Bay, Trinity Peninsula;
c) Mikkelsen Harbor, Trinity Island;
d) Union Glacier, Ellsworth Mountains.

Back-trajectory analysis suggests that long-range transport (from Patagonia, for example) has only a minor role as a source of light-absorbing aerosols in Antarctica.

**Supplementary Table 1**. Analysis of Variance (ANOVA) results showing the statistically significantly different site groupings for measurements of the BC concentration and the Ångström exponent shown in Fig. 2. Outputs show degrees of freedom (DF), F static and associated p value (Prob>F). Site Grouping 1 = Union Glacier. Grouping 2 = Deception Island, Livingstone Island, Trinity Island and Trinity Peninsula. Grouping 3 = Arctowski, Charlotte, Cuverville, Doumer, Collins Glacier, Maxwell Bay, Petermann, Detaille, Robert, Greenwich, Halfmoon.

| Source | DF | Sum of Squares | Mean Square | F Ratio | Prob > F |
|---|---|---|---|---|---|
| **Black Carbon:** | | | | | |
| Site groups | 2 | 110.8 | 55.4 | 23.7 | <0.0001 |
| Error | 119 | 278.0 | 2.3 | | |
| Total | 121 | 388.9 | | | |
| **Angstrom:** | | | | | |
| Site groups | 2 | 6.6 | 3.3 | 17.4 | <0.0001 |
| Error | 119 | 22.3 | 0.2 | | |
| Total | 121 | 28.9 | | | |

**Supplementary Table 2**. Tukey's Honestly Significant Difference Test outputs showing pair wise comparisons between the site groups (Fig. S2). The 3 site groupings are significantly different from each other for BC and only group 1 (Union Glacier) is significantly different for Ångström values. Site Grouping 1 = Union Glacier. Grouping 2 = Deception Island, Livingstone Island, Trinity Island and Trinity Peninsula. Grouping 3 = Arctowski, Charlotte, Cuverville, Doumer, Collins Glacier, Maxwell Bay, Petermann, Detaille, Robert, Greenwich, Halfmoon.

| Group | Comparison group | Difference | Std Err Dif | Lower CL | Upper CL | p-value |
|---|---|---|---|---|---|---|
| *Black Carbon:* | | | | | | |
| 2 | 1 | 2.5 | 0.4 | 1.6 | 3.3 | <0.0001 |
| 2 | 3 | 1.4 | 0.3 | 0.7 | 2.2 | <0.0001 |
| 3 | 1 | 1.0 | 0.3 | 0.2 | 1.8 | 0.0106 |
| *Angstrom:* | | | | | | |
| 2 | 1 | 0.6 | 0.1 | 0.3 | 0.8 | <0.0001 |
| 3 | 1 | 0.5 | 0.1 | 0.3 | 0.7 | <0.0001 |
| 2 | 3 | 0.1 | 0.1 | -0.1 | 0.3 | 0.6224 |

**Supplementary Table 3**. Black Carbon (BC) (units ng/g) minimum and maximum, 25% and 75% quartile, and median, reported for each of the site groupings (Fig. S2). Site Grouping 1 = Union Glacier. Grouping 2 = Deception Island, Livingstone Island, Trinity Island and Trinity Peninsula. Grouping 3 = Arctowski, Charlotte, Cuverville, Doumer, Collins Glacier, Maxwell Bay, Petermann, Detaille, Robert, Greenwich, Halfmoon.

| | | | |
|---|---|---|---|
| Grouping 1 | 0.00% | minimum | 0.6 |
| | 25.00% | quartile | 1.2 |
| | 50.00% | median | 1.9 |
| | 75.00% | quartile | 2.6 |
| | 100.00% | maximum | 3.8 |
| Grouping 2 | 0.00% | minimum | 0.9 |
| | 25.00% | quartile | 3.0 |
| | 50.00% | median | 4.4 |
| | 75.00% | quartile | 5.0 |
| | 100.00% | maximum | 8.1 |
| Grouping 3 | 0.00% | minimum | 0.6 |
| | 25.00% | quartile | 1.7 |
| | 50.00% | median | 2.9 |
| | 75.00% | quartile | 3.8 |
| | 100.00% | maximum | 8.0 |

**Supplementary Table 4**. Black Carbon (BC) (units ng/g) mean and standard deviation of each site grouping (Fig. S2). One-way t-tests are also reported to evaluate that the BC values of each grouping are significantly different to 0 and 1, respectively. Site Grouping 1 = Union Glacier. Grouping 2 = Deception Island, Livingstone Island, Trinity Island and Trinity Peninsula. Grouping 3 = Arctowski, Charlotte, Cuverville, Doumer, Collins Glacier, Maxwell Bay, Petermann, Detaille, Robert, Greenwich, Halfmoon.

| | Mean | Std Dev | Hypothesized mean | T-test statistic | Degrees of freedom | p-value |
|---|---|---|---|---|---|---|
| Grouping 1 | 1.9 | 0.8 | 0 | 13.1 | 32 | <0.0001 |
| | | | 1 | 6.3 | 32 | <0.0001 |
| Grouping 2 | 4.4 | 1.9 | 0 | 14.2 | 38 | <0.0001 |
| | | | 1 | 10.9 | 38 | <0.0001 |
| Grouping 3 | 2.9 | 1.5 | 0 | 13.7 | 49 | <0.0001 |
| | | | 1 | 9.0 | 49 | <0.0001 |
